# Transcriptional condensates: a blessing or a curse for gene regulation?

**DOI:** 10.1038/s42003-024-05892-5

**Published:** 2024-02-16

**Authors:** Martin Stortz, Diego M. Presman, Valeria Levi

**Affiliations:** 1 Instituto de Química Biológica de la Facultad de Ciencias Exactas y Naturales (IQUIBICEN), CONICET-Universidad de Buenos Aires, Buenos Aires, C1428EGA Argentina; 2 Center for Cancer Research, National Cancer Institute, National Institutes of Health, Bethesda, MD 20892 USA; 3 Instituto de Fisiología, Biología Molecular y Neurociencias (IFIBYNE), CONICET-Universidad de Buenos Aires, Facultad de Ciencias Exactas y Naturales, Buenos Aires, C1428EGA Argentina; 4 Departamento de Fisiología, Biología Molecular y Celular, Facultad de Ciencias Exactas y Naturales, Universidad de Buenos Aires, Buenos Aires, C1428EGA Argentina; 5 Departamento de Química Biológica, Facultad de Ciencias Exactas y Naturales, Universidad de Buenos Aires, Buenos Aires, C1428EGA Argentina

**Keywords:** Transcription, Nuclear organization

## Abstract

Whether phase-separation is involved in the organization of the transcriptional machinery and if it aids or inhibits the transcriptional process is a matter of intense debate. In this Mini Review, we will cover the current knowledge regarding the role of transcriptional condensates on gene expression regulation. We will summarize the latest discoveries on the relationship between condensate formation, genome organization, and transcriptional activity, focusing on the strengths and weaknesses of the experimental approaches used to interrogate these aspects of transcription in living cells. Finally, we will discuss the challenges for future research.

## Abstract (web summary)

In this Mini Review, the authors discuss the relationship between condensate formation, genome organization, and transcriptional activity, focusing on the experimental evidence behind the role of transcriptional condensates in gene regulation.

## Introduction

The mechanisms behind gene transcription have puzzled researchers for decades. Transcription factors (TFs) are central actors in this process since they bind to regulatory elements^1^ triggering the recruitment of coactivators, chromatin remodelers, the Mediator complex, and RNA polymerase II (Pol II). The first textbook models of transcription were built considering the sequential, hierarchical formation of these macromolecular protein-DNA complexes through lock-and-key interactions^2^, but these models were insufficient to describe many aspects of transcription in cells.

One of the most perplexing observations is that the diffusion of TFs within the nucleus is not sufficient to explain their velocity in locating specific DNA targets within the entire genome^3–5^. Moreover, a wealth of evidence shows that TF-chromatin interactions are highly dynamic, with lifetimes spanning from less than a second to minutes^6^, in contrast to the traditional view of transcriptional complexes bound to chromatin for hours^7^. Puzzlingly, the stochastic and apparently inefficient TF-chromatin interactions result in sustained transcriptional bursts that can last significantly longer^8^ and are the consequence of many regulatory processes that involve TF binding to enhancers, enhancer looping, chromatin remodeling, and Pol II recruitment and activation through mechanism(s) not completely elucidated^9,10^.

Although it was widely known that liquid-liquid phase-separation (LLPS) contributes to many emergent properties of biological systems, including cellular organization^11^, a tsunami of reports in the last few years^12^ proposed that the selective phase-separation of TFs and other related molecules near target genes contributes to transcriptional regulation^13^. In this review, we will explore the different models explaining the nature and function of these condensates, their link with chromatin organization, and the experimental evidence both in favor and against their role in transcription.

## Transcriptional hubs, condensates, or factories?

The discovery of membrane-less compartments within the nucleus dates from the nineteenth century with the foundational observations of the nucleolus^14^. Decades later, different electron microscopy techniques provided high-resolution views of the nuclear ultrastructure and its enormous variety of nuclear bodies with different functions (reviewed elsewhere^15^). In parallel, the improvement of fluorescence microscopy tools allowed the exploration of the intracellular distribution of biomolecules in live cells, leading to the observation that many actors involved in transcription do not distribute homogeneously throughout the nucleus but concentrate in distinct foci. In this section, we will discuss the different ideas aimed at explaining this heterogeneous organization. These models have granted different functions and names to these foci and still present challenges to be addressed.

A couple of decades ago, the observation of Pol II and active transcription sites concentrating as discrete foci within the nucleus of interphase cells led to the idea of transcription occurring within certain nuclear regions referred to as transcription factories (reviewed elsewhere^16^). This model—that contrasted with the classic idea of individual polymerases moving along an immobile DNA template^17^—postulates that Pol II molecules accumulate in stable clusters that physically interact with mobile chromatin loops to be transcribed^18–20^. Transcription factories may explain the transcription of some but not all genes, and consequently, they probably coexist with other modes of transcription^21^. In fact, the observation of Pol II molecules moving along very long and highly expressed genes^22^ suggests a more complex scenario than a static transcriptional platform.

Deeper scrutiny of transcription foci highlighted that they may concentrate many transcription-related molecules that interact among them weakly and with genomic loci forming networks with lifetimes below the minute time scale^23^. These dynamic networks are thought to favor the protein-protein and DNA-protein interactions required for transcription and are usually referred to as transcriptional hubs^24^. The propensity of TFs and other transcriptional players to form hubs was attributed to a common, modular structure shared by these molecules. Particularly, eukaryotic TFs present well-folded DNA-binding domains that recognize their chromatin targets and transactivation domains that usually include intrinsically disordered regions (IDRs)^25^ which could interact transiently with the IDRs of Pol II, cofactors, and other TFs^26–28^. Thus, both DNA-binding and transactivation domains of TFs would be responsible for the interaction network implicated in transcriptional hubs.

In the same direction, weak, multivalent IDR-IDR interactions seem to promote the phase-separation of IDR-containing proteins, including Med1, Pol II^29^, TBP^30^, BRD4^26^, YAP^31^, and OCT4^27^ in aqueous solutions. These observations led to the idea that similar droplet-like structures of transcription-related molecules with hallmarks of liquid phases could form inside cells and could play a role in transcription regulation^26,32,33^. According to this idea, chromatin regions act as scaffolds recruiting TFs and related molecules; this initial seed would grow by the incorporation of many other multivalent molecules with IDRs through weak interactions, finally forming a liquid transcriptional condensate^34,35^. We should point out that the terms ‘hubs’ and ‘liquid transcriptional condensates’ are not precisely defined in the literature yet; some authors are using the term ‘hubs*’* to indicate that they are formed by a relatively lower number of molecules^23^ and do not specify the mechanism involved in their formation^33^. In contrast, it is assumed that liquid transcriptional condensates form by LLPS and include a larger number of molecules. In any case, the term ‘transcriptional condensate*’* is now used to invoke any nuclear compartment concentrating biomolecules involved in transcription (Pol II, TFs, coactivators). Additionally, transcriptional repressors can also form condensates promoting transcriptional repression^36^, suggesting that condensation would act by favoring the specific activity of the condensed biomolecules, either activators or repressors. In this Mini Review, we will use the term ‘transcriptional condensates’ as an operational, agnostic definition, without implying that these structures necessarily form by phase-separation.

A recent report shows that the charge patterns of IDRs define the selective partitioning of biomolecules into different condensates, with possible functional consequences^37^, including the segregation of different transcriptional programs into specific transcriptional condensates^38^. Moreover, different types of transcriptional condensates seem to control several steps of gene activation, including the formation of preinitiation complexes, transcriptional pausing and elongation, super-enhancer clustering, and mRNA splicing and processing^39,40^. In fact, the biochemical reactions occurring during transcription may modulate the relative composition and properties of these condensates. For example, Pol II CTD phosphorylation will drive the transition from initiation to elongation, and therefore, the exit of Pol II molecules from initiation condensates to splicing condensates^41^. Also, newly synthesized RNA might regulate condensate formation and dissolution^42^. These features would work as feedback mechanisms controlling the different steps throughout mRNA biosynthesis.

However, other authors are challenging whether phase separation is involved in the formation of transcriptional condensates, claiming that alternative theoretical frameworks that also include weak and transient interactions between multivalent biomolecules could explain many of the properties assigned to liquid condensates^43–46^. Scrutinizing the biophysical properties of transcriptional condensates is especially challenging given their sub-diffraction size and the complex network of players involved, which make it difficult to use the standard in vitro approaches for studies in cells. There have been a few attempts to utilize single-molecule tracking in live cells as a tool to test whether distinct diffusive behaviors can be observed inside and outside condensates as predicted for distinct phases, leading to diverse and sometimes contradictory conclusions^44,47,48^.

Hence, the jury is still out on whether sufficient evidence has been provided for LLPS involvement in the formation of transcriptional condensates.

### Chromatin organization under the phase-separation lens

Chromatin presents a hierarchical organization at different scales that span from the micron-sized territories occupied by each chromosome, sub-micron features as topologically associating domains (TADs) and loops^49^, to the recently characterized nano-domains^50^. Although it is now widely accepted that many aspects of this organization are intimately related to genome function^51^, the relationship is still far from being understood^49^. Complementary approaches^52–55^ focusing on the physical properties of this biopolymer and/or its biochemical features, provided some clues regarding chromatin folding in the nucleus and its relevance to its functions. Chromatin-chromatin interactions are invoked as one of the major forces driving genome self-organization, defining domains and compartments, among other features^56^. At a sub-micron spatial scale, active processes such as loop extrusion drive the formation of loops and TADs and, combined with the passive, local exploration of the chromatin chain by diffusion, allow the interactions between relevant transcriptional regulatory elements, including enhancers and promoters^55,56^.

In addition, many aspects of chromatin organization have been analyzed under the phase-separation framework^52,57^. For example, reports indicate that chromatin behaves as a liquid phase in vitro^58^ and at the sub-micrometer/sub-second scales in cells^59^, whereas it behaves as a solid phase at the micrometers/minutes scales and also in live cells^59^. Additionally, the formation of constitutive and facultative heterochromatin domains might be produced by the phase-separation of associated heterochromatin proteins^60,61^, exemplifying the relevance of condensation to genome function.

**Fig. 1:**
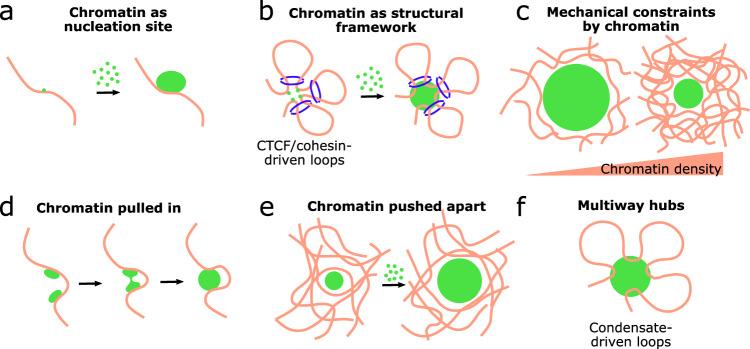
Proposed mechanisms involved in the interplay between nuclear condensates and chromatin organization. Illustrations of chromatin (pink) and condensates (green) schematically represent the ways they can influence each other. **a** Chromatin can act as a nucleation site for condensate formation. **b** Chromatin structure driven by CTCF-cohesin loops (cohesin complexes represented in blue) can act as a structural framework favoring condensate formation. **c** Chromatin microenvironment can exert mechanical forces on nuclear condensates. Local microenvironments would generate greater forces impairing condensate growth. **d** Condensate formation and growth can pull in two distant chromatin regions, driving chromatin looping. **e** Condensate growth can exert mechanical forces on surrounding chromatin, pushing it apart. **f** Condensate can bring multiple chromatin regions together, forming a multi-way chromatin hub.

More broadly, given that the structure of the genome influences its related functions^56^, we can expect an analogous interplay between transcriptional condensates and genome organization; this means that the former might shape the latter and vice versa (Fig. 1). In this sense, chromatin could act as a scaffold recruiting multivalent transcription-related molecules and triggering the formation of transcriptional condensates (Fig. 1a). From a chemical point of view, chromatin provides an adequate surface to promote the formation of condensates even at lower concentrations of the engaging molecules than those expected for a standard phase-separation process^62^. Given that transcriptional condensates may involve the interactions between multiple, distant chromatin regions, the 3D genome architecture can also contribute to condensate formation providing an adequate topological framework. In this sense, it has been shown that CTCF-dependent chromatin loops recruit Pol II, BRD4, and Mediator molecules, driving transcriptional condensate formation^63^ (Fig. 1b). In addition, the chromatinic microenvironment of condensates seems to modulate their shape and size (Fig. 1c), as very dense environments could limit the growth and coalescence of synthetic condensates^64^ while low-density chromatin regions would favor their formation^65^. However, the functional consequences of this potential modulation remain elusive.

Conversely, nuclear condensates themselves can reorganize the local chromatin architecture^66^. For example, it has been shown that TFs such as FOXA1 and SOX2 co-condense with DNA in vitro, exerting a tension force on the DNA strand which can bring together distal regions^67,68^. This force observed in vitro could contribute to chromatin looping associated with transcription regulation. Thus, chromatin-sticky condensates may bring together genomic loci through coalescence involving capillary forces^65,69^ (Fig. 1d), whereas, in other cases, the growth of a non-sticky condensate may exclude chromatin, pulling apart certain genomic loci^65^ (Fig. 1e).

Although further work needs to be done to demonstrate that many of these processes observed in vitro or with synthetic condensates in cells also occur in vivo, several studies suggest that transcriptional condensates could regulate gene expression by modulating genome structure. Specifically, the formation of phase-separated condensates of certain TFs has been linked to a 3D rearrangement of the genome and the consequent activation of target genes as observed during somatic cell reprogramming^70^, B-cell to macrophage trans-differentiation^71^ and leukemogenesis^72^. The use of synthetic TFs capable of phase-separate also illustrates how transcriptional condensates targeted to specific loci could increase chromatin accessibility, promote long-range contacts between genes and regulatory regions, and induce transcription of these target genes^66^.

Transcriptional condensates have also been postulated as responsible for the formation of multi-way hubs comprising many genes involved in similar biological functions (Fig. 1f). This is the case for housekeeping genes in mammalian cells^73^ wherein the contacts between their promoters are driven by the TF Ronin. Similarly, a report claims that HSF1 is responsible for the coalescence of long-range, distal genes responding to heat-shock stress in yeast^74^. These examples illustrate additional ways in which transcriptional condensates may contribute to chromatin architecture, thus modulating gene expression.

### A functional link between transcriptional condensates and transcriptional activity: evidence and caveats

In the previous sections, we discussed some ideas on the nature of transcriptional condensates and reviewed evidence linking them with chromatin organization. The concepts presented above point to the inevitable relevant question of what the function of these condensates in transcriptional control might be. In this last section, we will present and discuss the evidence either in favor of or against the role of transcriptional condensates in transcriptional regulation. However, interpreting the complex and sometimes apparently contradictory spectrum of evidence requires first understanding the strengths and limitations of the different strategies usually employed to address this question. In Table 1, we provide a representative overview of the current literature describing the biological models, experimental approaches, and relevant results from key studies in the field.

**Table 1:** Role of transcriptional condensates on transcriptional activity: evidence and experimental strategies

Cell lines or organisms (live/fixed)	TF or transcription-related molecule	Strategy (tools)	Role of condensates in transcription	Key results and conclusions	Ref.
Stem cells (fixed)	Natural TFs	Correlative (colocalization)	Activation	TFs form condensates with Med1 through their activation domains.	^27^
Stem cells (live and fixed)	Natural TFs	Correlative (colocalization)	Activation	Condensates of a coactivator and Med1 form at super-enhancers.	^26^
Cancer cells (live)	Synthetic coactivator + MS2 reporter gene	Correlative (colocalization); Causal (pharmacology, optogenetics)	Activation	1) Coactivator forms condensates with Pol II CTD; 2) light-induced condensates amplify transcription.	^39^
Cancer cells (live and fixed)	Negative transcriptional regulator	Correlative (condensates visualization and gene expression assays); Causal (domain swapping)	Inhibition	1) Natural IDR is required for heat-shock-dependent condensate formation and transcriptional repression; 2) other IDRs promote condensate formation and gene repression independently of heat-shock.	^36^
Stem and cancer cells (live and fixed)	Natural and synthetic TFs + PP7 reporter gene	Correlative (colocalization); Causal (domain swapping, optogenetics)	Activation	1) TFs and p300 form condensates through IDR-IDR interactions; 2) p300 is acetylated (active) at condensates; 3) condensates modulate initiation rate and burst duration.	^80^
Immortalized cells and adult mice (live and fixed)	Synthetic TFs + enzymatic reporter gene	Causal (domain swapping, optogenetics)	Activation	1) IDR promote formation of synthetic TF condensates; 2) light-induced TF condensates increase transcription.	^81^
Immortalized cells and primary cultures (live and fixed)	Onco-fusion and synthetic TFs	Causal (domain swapping, deletions, mutations pharmacology)	Activation	1) Onco-fusion TF IDR promotes formation of condensates, chromatin looping, oncogenes activation and oncogenesis; 2) swapping with other IDRs also promotes condensate formation with similar oncogenic properties.	^87^
Cancer cells (fixed)	Natural coactivator	Correlative (colocalization)	Inhibition	p300 coactivator condensates colocalize with repressive histone marks.	^104^
Cancer cells (live and fixed)	Onco-fusion TF	Causal (competition with exogenous IDRs)	Inhibition	1) Excess in homotypic IDR-IDR interactions represses onco-fusion TF-driven transcription; 2) larger condensates formed by heterotypic IDR-IDR interactions exert more severe transcriptional repression.	^23^
Cancer cells (live and fixed)	Synthetic TFs + MS2 reporter gene	Causal (domain swapping, optogenetics)	None / Inhibition	1) Strength of TFs activation domains correlates with their propensity to form condensates; 2) condensate formation per se does not enhance transcriptional activity; 3) increasing valency might inhibit transcription.	^43^
Cancer cells (live and fixed)	Natural chromatin reader	Correlative (colocalization); Causal (mutations, deletions, insertions)	Activation (only at endogenous levels)	1) Chromatin reader and oncogenic mutants form condensates at specific loci; 2) degree of condensation correlates with gene expression level; 3) overexpressed mutants form non-functional condensates.	^105^
Prostate-derived cells (live and fixed)	Natural TF	Correlative (colocalization) Causal (pharmacology, mutations, truncations, optogenetics)	Activation	1) Ligand-activation triggers the formation of TF condensates with Med1, H3K27ac, and Pol II Ser-5-P; 2) a small inhibitor compound targeting TF IDR disrupts TF condensates.	^78^
Cancer and epithelial cells (live and fixed)	Synthetic TFs + PP7 reporter gene	Correlative (colocalization); Causal (pharmacology, domain swapping)	Activation	1) TF clustering propensity modulates gene expression; 2) only 4% of TF condensates colocalize with nascent RNA sites.	^82^
Cancer cells (fixed)	Natural TF	Correlative (colocalization); Causal (mutations)	Activation	1) Heat-shock stimulation triggers the formation of TF condensates at targeted loci; 2) TF condensates include cofactors, Pol II, and H3K4me3; 3) a mutant TF that does not form condensates presents impaired binding to chromatin and lower activity.	^79^
Immortalized and cancer cells (live and fixed)	Synthetic TFs	Causal (mutations, truncations)	Activation	1) TF condensate formation increases chromatin accessibility and looping at targeted genes and is linked to gene activation; 2) proteomic analysis identifies Pol II, other TFs, coactivators, Mediator, and chromatin remodelers at condensates.	^66^
*Drosophila* embryos (live cells)	Synthetic TF + MS2 reporter gene	Correlative (condensates visualization and gene expression assays); Causal (mutations, insertions)	Activation	1) Formation of TF condensates at enhancers correlates with transcriptional bursting; 2) longer synthetic IDRs increase RNA production, but there is a sweet spot for optimal transcription; 3) TF clustering favors the interactions between distal enhancer and promoter.	^83^
Yeast (live)	Natural TF + PP7 reporter gene	Causal (truncations, mutations)	Neutral or inhibitory	1) TF form condensates at endogenous target genes; 2) both IDRs and DBD contribute to but are not essential for condensate formation; 3) the activity of a reporter gene does not depend on the presence of a colocalized condensate; 4) a DBD-mutant TF recruited to condensates does not contribute to transcriptional activation and may actually inhibit transcription.	^84^
Immortalized and cancer cells (live and fixed)	Natural and synthetic TFs	Casual (truncations, mutations, pharmacological)	Activation or inhibition (fine tuning of optimal IDR interactions)	1) TF IDR is necessary but not sufficient for condensate formation; 2) swapping IDRs has different effects on condensate formation and transcriptional activity; 3) poly-glutamine track extension within the IDR favors condensate formation but decreases transcriptional activity.	^95^
Cancer cells (live)	Natural TFs	Causal (mutations, truncations)	Inhibition	TF with an additional IDR increases transcription below critical concentration (no condensate formation) but decreases transcription when forming condensates at higher concentrations	^106^
Mice liver (fixed), cancer cell lines (live) and mice (live?)	Natural TF	Correlative (colocalization); Causal (truncations)	Inhibition	1) Donut-shape condensates containing TF and corepressor; 2) IDR necessary for condensation and transcriptional repression.	^107^

The Table summarizes some of the main evidence in the literature regarding the role of transcriptional condensates in regulating transcriptional activity, focusing on the experimental tools and strategies utilized. Only those experiments performed on cultured cells or organisms were included (evidence obtained from in vitro approaches was excluded from this table). *TF* transcription factor, *CTD* C-terminal domain, *IDR* intrinsic disordered region, *PP7/MS2* RNA labeling system to track transcription in real time, *Pol II Ser-5-P* post-translational modification of Pol II that indicates transcription initiation, *DBD* DNA-binding domain.

**Fig. 2:**
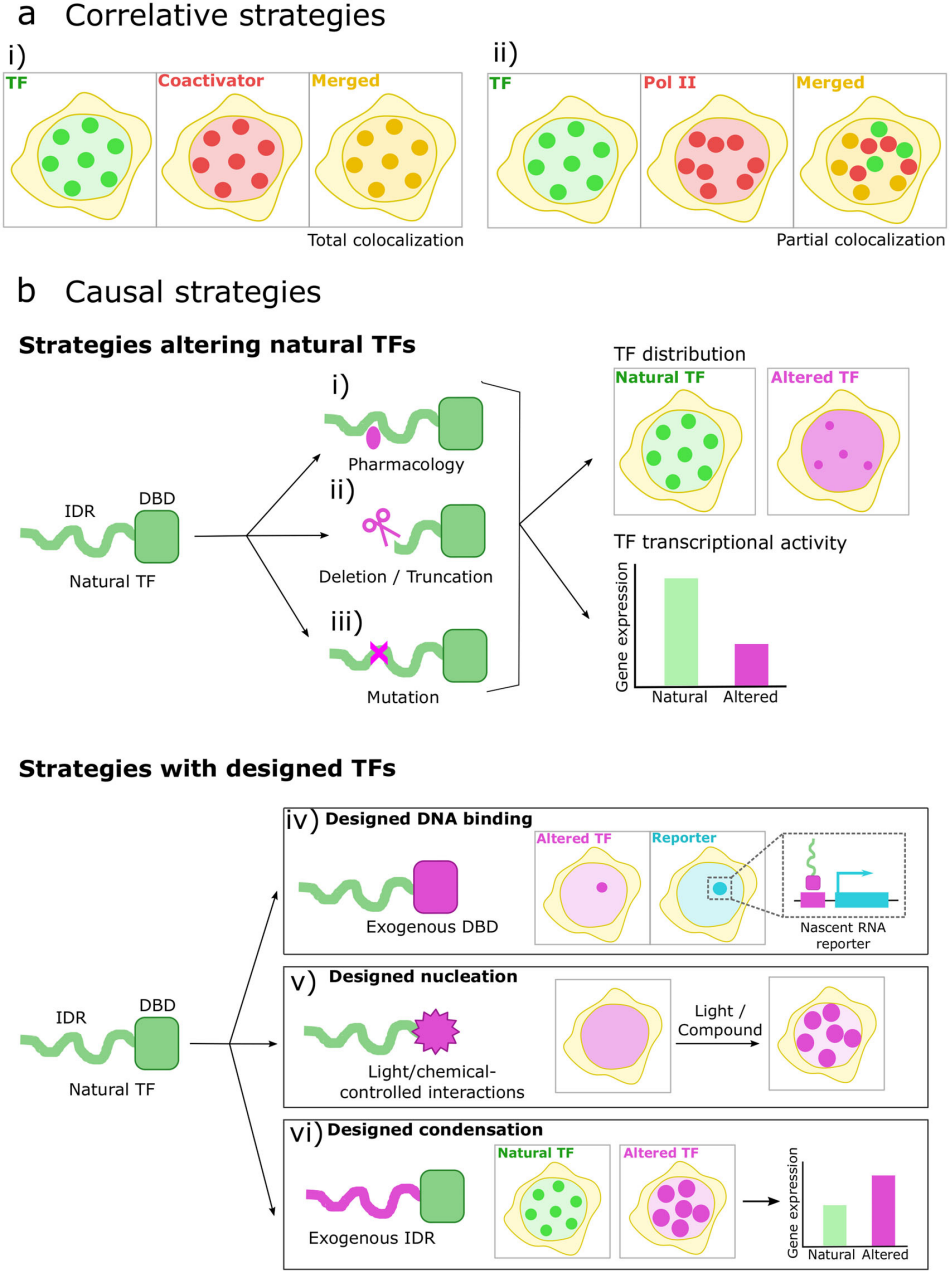
Strategies to study the role of transcriptional condensates on transcriptional activity. **a** *Correlative strategies* generally involve using fluorescence microscopy to compare the spatial distributions of the condensates of a TF of interest (green) with those of other biomolecules with a known role in transcription, such as coactivators or Pol II (red). The merged images illustrate different degrees of colocalization (yellow): **i**) ‘total colocalization’, where both biomolecules colocalize in the same condensates, and **ii**) ‘partial colocalization’, where only a subset of condensates colocalize with the condensates formed by the other biomolecule. **b** *Causal strategies* involve performing perturbations of TF structure and analyzing their impact on certain properties in the cells. Top panels: TFs usually present a modular structure with a DNA-binding domain (DBD), and an IDR responsible for transactivation, target searching, and, often, condensate formation. TF structure can be experimentally altered by different approaches, including **i**) pharmacological targeting (oval), **ii**) deletions/truncations (scissors), and **iii**) point-mutations (cross). Analyzing the TF spatial distribution and the TF-driven transcriptional activity after these perturbations allows for exploring the contribution of the specific TF structural features to condensate formation and activity. Bottom panels: *Synthetic TFs* (green-purple) can be designed by fusing domains of the TF of interest to moieties with known properties. **iv**) *Designed DNA binding*: replacing the TF DBD with a well-studied, bacterial TF DBD, or RNA-guided dCas9 (not illustrated), and introducing a specific transcription reporter (cyan) driven by this DBD allow studying how the TF IDR contributes to transcriptional activity at these designed DNA targets. **v**) *Designed nucleation*: replacing the TF DBD with a responsive module capable of light- or chemical-controlled oligomerization helps to understand the contribution of the TF IDR to condensate formation. **vi**) *Designed condensation*: this strategy includes analyzing the effects of replacing the TF IDR with an exogenous IDR with enhanced condensation propensity, helping to understand the role of condensation on the TF activity at its natural DNA targets. These strategies can also be combined or modified to analyze more complex scenarios, according to the specific system.

One important experimental caveat in all these studies is how to selectively affect the ability of a transcriptional player to form condensates without altering other important activities. For example, IDRs of TFs and coactivators appear essential for condensate formation in many cases^27^, but they are also linked to activation functions^25^ and even to the DNA target searching process^75,76^. Therefore, it is extremely difficult to discriminate among the many functions IDRs can have on transcriptional regulation, including biomolecular condensation. This important limitation makes it relevant to understand the methodological aspects of the specific studies; Fig. 2 summarizes recent and commonly used experimental strategies designed to test whether transcriptional condensates are involved in transcriptional regulation.

Correlative studies (Fig. 2a) often include the use of different fluorescence microscopy techniques and labeling strategies to analyze the colocalization of nuclear condensates and key molecular players in transcriptional regulation, either in live or fixed cells. Noteworthy, fixation has been reported to produce artifacts and change the appearance of condensates^77^, therefore some colocalization experiments performed in fixed cells should be validated on live samples if possible. In the particular case of co-immunofluorescence studies, it is also imperative to perform additional experiments on live-cell systems and purified proteins to characterize a cellular object as a condensate.

Correlative reports have shown the colocalization between TFs condensates and other transcriptional players or epigenetic marks (Fig. 2a.i) such as Med1, BRD4, H3K27ac, and Pol II^27,35,78,79^. Even though, in many cases, the colocalization is partial (Fig. 2a.ii), at least a fraction of TF condensates appears to co-occupy, within the resolution limit of the techniques, sites of active transcription. Similarly, TF condensates were found to colocalize with target enhancers or target genes labeled by DNA-FISH^26,27^ and with fluorescently labeled reporter genes^39,43,80–84^. In addition, a recent study found that condensate proximity with a gene locus correlates with RNA burst enhancement^85^. Taken together, these results point to at least a population of transcriptional condensates being involved in transcription.

Causal studies (Fig. 2b), which means probing beyond correlation of a cause-effect link between transcriptional condensates and transcription, are intrinsically more difficult to achieve. There have been three main strategies used thus far: pharmacological/chemical treatments, deletions/truncations/mutations of key transcriptional players, and synthetic/chimera TFs coupled with reporter genes. Chemical manipulation (Fig. 2b.i) includes—but is not limited to—the use of aliphatic alcohols, which, in principle, perturb weak hydrophobic interactions, resulting in the dissolution of some liquid condensates^86^. Even though this behavior was traditionally considered the fingerprint of LLPS and this dissolution correlated with decreased transcriptional activity^26,27,79,80,87^, nowadays, this treatment is considered to have many confounding effects on cells beyond affecting condensates^88–90^. Nevertheless, it might still be useful to explore the chemical properties of the intermolecular interactions involved in condensates rather than to establish evidence of phase separation^91^.

While pharmacological studies are scarce, there is an interesting report wherein a compound specifically targets the androgen receptor’s IDR, disrupting both the receptor condensates and its transcriptional activity^78^. Targeting the IDR of the oncogenic driver TF MYC also affects its transcriptional activity^92^, but the effect on MYC condensates has not been evaluated. Beyond a potentially suitable tool to causally link condensates to transcriptional activity, pharmacological manipulation of condensates can potentially be used for clinical applications^93^. We expect that high-throughput screening strategies will amplify the pharmacological toolkit to target IDRs and transcriptional condensates in the near future.

Deletions, truncations (Fig. 2b.ii), and point-mutations (Fig. 2b.iii) in different transcriptional players are suitable strategies to test causality, with many examples shown in Table 1. However, it is worth noting that given the likely lack of true modularity in TF domains^76^, the complex intertwined connections between a measured phenotype and untested pleiotropic effects of mutations make the lines between correlation and causality a bit blurry. For example, and as we pointed out above, deleting an IDR of a TF might affect its mechanism of action at different levels, making it difficult to discriminate which one is responsible for the change in the phenotypic output.

Synthetic/chimeric TFs have provided the most promising experimental evidence thus far to test the hypothesis of a causal link between transcriptional condensates and transcriptional activity (Table 1). Synthetic TFs usually contain a DNA-targeting moiety (such as a catalytically dead Cas9, or the DNA-binding domain of a eukaryotic/prokaryotic TF) (Fig. 2b.iv), an activation domain of interest, and an ’optogenetic’ domain (e.g., CRY2), which allows control of oligomerization and condensate formation in a switch-on/off fashion using light^39,43,66,80,81^ (Fig. 2b.v). These constructs may sometimes include additional IDRs (e.g., FUS IDR) to enhance their condensation propensity (Fig. 2b.vi). Many of these approaches are frequently combined with the use of reporter genes with specific binding sequences for the DNA-binding domain of the synthetic TF and stem-loops within the reporter nascent RNA that bind to coating proteins fused to fluorescent tags, providing a spatially resolved, readable transcriptional output (Fig. 2b.iv). The intrinsic artificial nature of these constructions is a valid concern, and therefore, the extrapolation of the results obtained from synthetic TFs with reporter genes to the biology of natural TFs in a native context remains unclear.

Many studies using synthetic/chimera TF strategies pointed to a positive functional role of condensates in transcriptional activity^39,66,80,81,83,87^ (Table 1). Nevertheless, a more complex picture is portrayed in a study wherein DNA binding and oligomerization are independently controlled within a synthetic TF. Here, the authors analyze how artificial TF clustering correlates with the transcriptional output of a fluorescent reporter gene, reporting the occurrence of transcription with and without clustering. Interestingly, only 4% of TF clusters colocalize with nascent RNA signals, with a wide spectrum of effects ranging from inhibition to sustained activation depending on the artificial TF^82^. In fact, other groups have argued that transcriptional condensates are either neutral or even inhibitory for transcription^23,43,84^. In this sense, a clean experimental design in yeast demonstrated that the activation of a reporter gene did not depend on clustered TF recruitment^84^.

Both subtle differences and, in some cases, opposite conclusions among studies on the role of condensates in transcription could be due to a plethora of factors. From cell-type to gene-specific effects and all the way to protein-specific action can explain the observed differences. Moreover, as with most biological processes, transcriptional condensates may have an ideal composition range for optimal activity wherein any excess or deficiency might have inhibitory effects, as illustrated by others^23,94,95^, and this optimal concentration ratio could be system- and/or condition-specific.

## Outlook/perspectives

Over the last few years, we witnessed striking adjustments in how we understand transcription. One of the most stunning changes is the multi-phase picture of the nucleus^96^, where some of the membrane-less subnuclear compartments concentrate transcriptional actors interacting through weak, multivalent bonds. Although substantial work has been done to elucidate whether these subnuclear structures play a role in the spatial and/or temporal control of transcription, we are still missing fundamental pieces to understand this complex transcriptional puzzle (Box 1).

One of the most relevant challenges to explain the relatively slow progress in this specific topic is the absence of adequate tools to study these nuclear compartments. Particularly, there is not a consensus in the scientific community on the evidence required to firmly assess LLPS involvement in the formation of a given type of condensates in cells (discussed in detail elsewhere^44,97,98^). Because of these technical difficulties, probing a functional relationship between biomolecular condensation and transcriptional regulation is still very challenging^13^. A major criticism in the field is that many observations performed in vitro or by the artificial manipulation of proteins to generate condensates in cellular systems cannot be extrapolated to native proteins in physiological conditions. Forcing local molecular concentrations higher than their physiological values may favor non-specific interactions between multivalent molecules and the formation of abnormal condensates^99^. Moreover, chemical cell fixation might promote the aberrant distribution of many nuclear biomolecules^77,100,101^, perturb chromatin organization^101,102^, and underestimate the transient nature of relevant interactions, overall preventing the straightforward interpretation of studies using fixed cells. In the same direction, it might be risky attempting to directly correlate live, single-cell imaging observations with those obtained from experiments based on the chromosome conformation capture procedure, a method based on crosslinking chromatin regions that provides information about the three-dimensional organization of the genome^103^.

Despite these limitations, growing evidence points toward a functional link between (some) transcriptional condensates and transcriptional regulation (Table 1). The state-of-the-art data suggests that this relationship is more complex than the initial view of transcriptional condensates concentrating transcription-activating molecules near gene promoters and thus promoting transcription. Indeed, the small sample of key reports compiled in this Mini Review attempts to be representative of the abundant literature in the field, ruling out a univocal relation between condensates and transcription regulation.

Our unresolved transcriptional puzzle suggests that transcriptional condensates with different compositions and functions may coexist in a single cell and be part of another temporal/spatial regulatory layer of transcription. Biomolecular condensates may be directly engaged in this regulation or could also contribute to shape chromatin structure at different scales, thus indirectly modulating gene expression.

Box 1 Questions and challenges looking forward **Outstanding questions and challenges**

- Is condensation functionally involved in transcriptional initiation? The field is still lacking a definitive roadmap to demonstrate that certain membrane-less bodies are formed via biomolecular condensation, and the tools to separate condensation from other functions of the same protein.
- It is hard to correlate live-cell microscopy measurements with the outputs of high-throughput genomics. Beyond the inherent difference in the experimental unit (single-cell vs average population measurements), fixation seems to severely affect chromatin organization/folding^77^ and condensate formation^108^.
- Who comes first, chromatin organization or transcriptional condensates? Does chromatin rule, is it the other way around, or does it depend on each case? Can we think of condensates or even smaller hubs to play a function as local regulators of chromatin accessibility?
- How can we mechanistically explain the functional outcomes associated with condensates built from different constituents? The intertwined different functions of TFs IDRs make it particularly challenging to answer this question.
- Does a given family of transcriptional condensates within a single cell present different compositions? Is this heterogeneity relevant to the transcriptional output?
